# A Signature of Maternal Anti-Fetal Rejection in Spontaneous Preterm Birth: Chronic Chorioamnionitis, Anti-Human Leukocyte Antigen Antibodies, and C4d

**DOI:** 10.1371/journal.pone.0016806

**Published:** 2011-02-04

**Authors:** JoonHo Lee, Roberto Romero, Yi Xu, Jung-Sun Kim, Vanessa Topping, Wonsuk Yoo, Juan Pedro Kusanovic, Tinnakorn Chaiworapongsa, Sonia S. Hassan, Bo Hyun Yoon, Chong Jai Kim

**Affiliations:** 1 Perinatology Research Branch, National Institute of Child Health and Human Development, National Institutes of Health, Department of Health and Human Services, Bethesda, Maryland, and Detroit, Michigan, United States of America; 2 Department of Obstetrics and Gynecology, Wayne State University School of Medicine, Detroit, Michigan, United States of America; 3 Center for Molecular Medicine and Genetics, Wayne State University, Detroit, Michigan, United States of America; 4 Department of Pathology, Samsung Medical Center, Sungkyunkwan University School of Medicine, Seoul, Republic of Korea; 5 Translational Research and Clinical Epidemiology, Department of Internal Medicine, Wayne State University School of Medicine, Detroit, Michigan, United States of America; 6 Department of Obstetrics and Gynecology, Sótero del Rio Hospital, Santiago, Chile; 7 Department of Obstetrics and Gynecology, School of Medicine, Pontificia Universidad Católica de Chile, Santiago, Chile; 8 Department of Obstetrics and Gynecology, Seoul National University College of Medicine, Seoul, Republic of Korea; 9 Department of Pathology, Wayne State University School of Medicine, Detroit, Michigan, United States of America; Chinese Academy of Sciences, China

## Abstract

**Background:**

Chronic chorioamnionitis is found in more than one-third of spontaneous preterm births. Chronic chorioamnionitis and villitis of unknown etiology represent maternal anti-fetal cellular rejection. Antibody-mediated rejection is another type of transplantation rejection. We investigated whether there was evidence for antibody-mediated rejection against the fetus in spontaneous preterm birth.

**Methods and Findings:**

This cross-sectional study included women with (1) normal pregnancy and term delivery (n = 140) and (2) spontaneous preterm delivery (n = 140). We analyzed maternal and fetal sera for panel-reactive anti-HLA class I and class II antibodies, and determined C4d deposition on umbilical vein endothelium by immunohistochemistry. Maternal anti-HLA class I seropositivity in spontaneous preterm births was higher than in normal term births (48.6% vs. 32.1%, p = 0.005). Chronic chorioamnionitis was associated with a higher maternal anti-HLA class I seropositivity (p<0.01), significant in preterm and term birth. Villitis of unknown etiology was associated with increased maternal and fetal anti-HLA class I and II seropositivity (p<0.05, for each). Fetal anti-HLA seropositivity was closely related to maternal anti-HLA seropositivity in both groups (p<0.01, for each). C4d deposition on umbilical vein endothelium was more frequent in preterm labor than term labor (77.1% vs. 11.4%, p<0.001). Logistic regression analysis revealed that chronic chorioamnionitis (OR = 6.10, 95% CI 1.29–28.83), maternal anti-HLA class I seropositivity (OR = 5.90, 95% CI 1.60–21.83), and C4d deposition on umbilical vein endothelium (OR = 36.19, 95% CI 11.42–114.66) were associated with preterm labor and delivery.

**Conclusions:**

A major subset of spontaneous preterm births has a signature of maternal anti-fetal cellular and antibody-mediated rejections with links to fetal graft-versus-host disease and alloimmune reactions.

## Introduction

Preterm birth is the leading cause of perinatal mortality and morbidity worldwide [1]. Moreover, the rate of preterm birth has been rising in most developed countries, ranging from 5% to 13% of all deliveries [2], [3]. The socioeconomic impact of preterm birth cannot be overestimated, and the cost of preterm birth is $26 billion per year in the United States [4], [5]. While preterm birth is known to be associated with various obstetric disorders such as intra-uterine infection/inflammation and preeclampsia [1], [6], the causes and precise mechanisms are not fully understood. A robust clinico-pathologic classification of heterogeneous varieties of preterm birth is fundamental to the diagnosis, prognosis, and design of ideal therapeutic interventions. Therefore, the elucidation of the essential pathophysiology of different types of preterm birth is an urgent and important matter.

The fetus is the most successful semi-allograft. Maternal immune tolerance of the fetus, therefore, is essential for successful pregnancy [7], [8]. Accordingly, failure of maternal tolerance to the fetus has been suggested as a mechanism of adverse pregnancy outcomes: recurrent pregnancy loss, fetal growth restriction, and preeclampsia [9]–[11]. Allograft rejection involves both innate and adaptive immune mechanisms [12], [13]. The most important alloantigens, major histocompatibility complex (MHC) class I and class II molecules, comprise the human leukocyte antigen (HLA) system [12], [14]. Transplantation rejection is initiated after the recognition of donor alloantigens by recipient T cells via direct or indirect pathways, which is followed by both cellular (lymphocyte-mediated) and humoral (antibody-mediated) immune responses [12]. The presence of circulating anti-HLA antibodies in the recipient is a serious obstacle to successful organ transplantation [15], [16].

Chronic chorioamnionitis and villitis of unknown etiology of the placenta are closely related immunologic inflammatory lesions, harboring features of allograft rejection of the mother and graft-versus-host disease of the fetus [17], [18]. Chronic chorioamnionitis, defined as lymphocytic infiltration of the chorioamniotic membranes [19], is the most common lesion found in placentas of spontaneous preterm births although it can also be found in a small fraction (9%) of term births [18]. It is associated with a robust increase in the amniotic fluid T cell chemokine CXCL10 concentration and CXCL9, CXCL10, and CXCL11 mRNA expression in the chorioamniotic membranes [18]. The essential feature of this inflammation is chemotaxis of T cells expressing CXCR3, which is a receptor for CXCL9, CXCL10, and CXCL11. CXCR3 mediates both chemotactic and anti-angiogenic activities, and it is also expressed in natural killer cells and macrophages. A unique feature of chronic chorioamnionitis and villitis of unknown etiology is that fetal tissues - the chorioamniotic membranes and chorionic villi - are infiltrated by maternal T cells. Maternal origin of T cells in villitis of unknown etiology has been elegantly shown by *in situ* hybridization [20]. Given that chronic chorioamnionitis and villitis of unknown etiology are consistent with maternal anti-fetal cellular rejection, we hypothesized that maternal antibody-mediated rejection against the fetus could also be associated with preterm labor and preterm prelabor rupture of membranes. The clinical significance of anti-paternal HLA antibodies in pregnant women has been mainly studied in the settings of recurrent miscarriage but not in spontaneous preterm birth [21]–[27]. The potential significance of maternal antibody-mediated rejection against the fetus is two-fold as antibodies crossing the placenta can induce a systemic fetal alloimmune reaction as seen in Rh incompatibility and alloimmune thrombocytopenia [28], [29].

This cross-sectional study was conducted to examine the frequency and significance of anti-HLA antibodies in maternal and fetal sera according to the presence of chronic chorioamnionitis, particularly in the context of spontaneous preterm birth.

## Methods

### Study population

We studied 280 women with the following diagnoses: (1) normal pregnancy and term delivery (n = 140): term not in labor (n = 70) or term in labor (n = 70), and (2) spontaneous preterm delivery (n = 140): preterm labor with intact membranes (n = 70) or preterm prelabor rupture of membranes (n = 70). Fetal congenital anomalies and cases with small-for-gestational-age neonates were excluded from this study. Preterm labor was defined as the presence of regular uterine contraction associated with cervical dilatation, followed by delivery before 37 completed weeks of gestation. Preterm prelabor rupture of membranes was diagnosed by sterile speculum examination when pooling of amniotic fluid in the vagina occurred or when positive nitrazine and ferning tests, conducted when necessary, were confirmed before 37 weeks of gestation in the absence of labor. Placental tissues and cord blood samples were collected at the time of delivery, and maternal blood samples were drawn and collected within 7 days before and after delivery, which allowed us to maintain a meaningful temporal relationship between placental pathology and the status of anti-HLA antibodies in maternal and fetal sera. Samples were kept at −80°C until use.

All patients were Hispanic women who were enrolled and delivered at the Sótero del Rio Hospital in Santiago, Chile. Serum and tissue samples from all patients were retrieved from the Bank of Biological Materials of the Perinatology Research Branch, *Eunice Kennedy Shriver* National Institute of Child Health and Human Development, National Institutes of Health, U. S. Department of Health and Human Services. All women provided written informed consent before participating in the study. The use of clinical data and the collection and utilization of biological samples for research purposes were approved by the Institutional Review Boards of the Sótero del Rio Hospital, Santiago, Chile (an affiliate of the Pontificia Universidad Católica de Chile), Wayne State University, and the *Eunice Kennedy Shriver* National Institute of Child Health and Human Development, National Institutes of Health, U.S. Department of Health and Human Services.

### Placental pathology

Histopathological changes of the placenta were defined according to diagnostic criteria proposed by the Perinatal Section of the Society for Pediatric Pathology, which include lesions consistent with amniotic fluid infection, maternal vascular underperfusion, and fetal vascular thrombo-occlusive disease [30]. Diagnosis of villitis of unknown etiology was based on histologic criteria previously defined [17], and chronic chorioamnionitis was diagnosed when lymphocytic infiltration into the chorionic trophoblast layer or chorioamniotic connective tissue was present as described previously [18].

### Flow cytometry of panel-reactive anti-HLA antibodies

Flow cytometric analysis of panel-reactive anti-HLA class I and class II antibodies in maternal and cord blood sera was performed using the FlowPRA®-I screening test (One Lambda Inc., Canoga Park, CA, USA) and FlowPRA®-II screening test (One Lambda Inc.) according to the manufacturer's instructions. Class I or class II microbeads were mixed with 20 µl of serum, and incubated for 30 min at room temperature with gentle rotation. The beads were washed 3 times with 1 ml of Flow PRA wash buffer by centrifugation at 9,000×g for 2 min, followed by incubation with 100 µl of FITC-conjugated F(ab)2 fragment of Fcγ fragment specific goat anti-human IgG for 30 min. After washing the beads twice with 1 ml of wash buffer and adding 0.5 ml of fixing solution (PBS with 0.5% formaldehyde), the FL1 fluorescence of 5,000 events was analyzed using BD LSRII flow cytometry (BD Biosciences, San Jose, CA, USA). A sample with reactivity of 10% or more was considered positive for panel-reactive anti-HLA antibodies [31], [32].

### Immunohistochemistry

Immunohistochemistry assessed C4d deposition on umbilical blood vessels. Five-µm-thick sections of formalin-fixed, paraffin-embedded umbilical cords were placed on silanized slides and stained using a Ventana Discovery automatic staining system (Ventana Medical Systems, Tucson, AZ, USA). Immunostaining was performed using a mouse monoclonal anti-human C4d antibody (1∶100, ALPCO Diagnostics, Salem, NH, USA). The Discovery® DAB Map™ Kit (Ventana Medical Systems) detected the chromogen reaction of horseradish peroxidase.

### Statistical analysis

The characteristics were examined to find differences between women who had normal pregnancy and term delivery (n = 140) and women who had spontaneous preterm delivery (n = 140). For continuous variables, distributions were examined for normality using Kolmogorov-Smirnov tests. When data were far from normality, the Mann-Whitney U test was performed between groups. If there was normality in continuous variables, an unpaired t-test was used. For categorical variables, proportions were compared with Fisher's exact test or *χ* ^2^ test. Means and standard errors of mean (SEMs) were reported for continuous variables whereas frequencies and percentages were calculated for categorical variables. The Spearman rank correlation test determined the relationship between maternal and fetal anti-HLA antibodies. Logistic regression analysis was performed to determine (1) the relative effect of clinical variables and placental pathology on maternal and fetal anti-HLA seropositivity and (2) the effect of placental pathology, anti-HLA antibodies in maternal and fetal sera, and C4d deposition on the occurrence of spontaneous preterm birth. Statistical analyses were performed using SPSS Version 15.0 (SPSS, Inc., Chicago, IL, USA). All p values are two-sided, and a value of p<0.05 is considered to be statistically significant.

## Results

**Figure 1:**
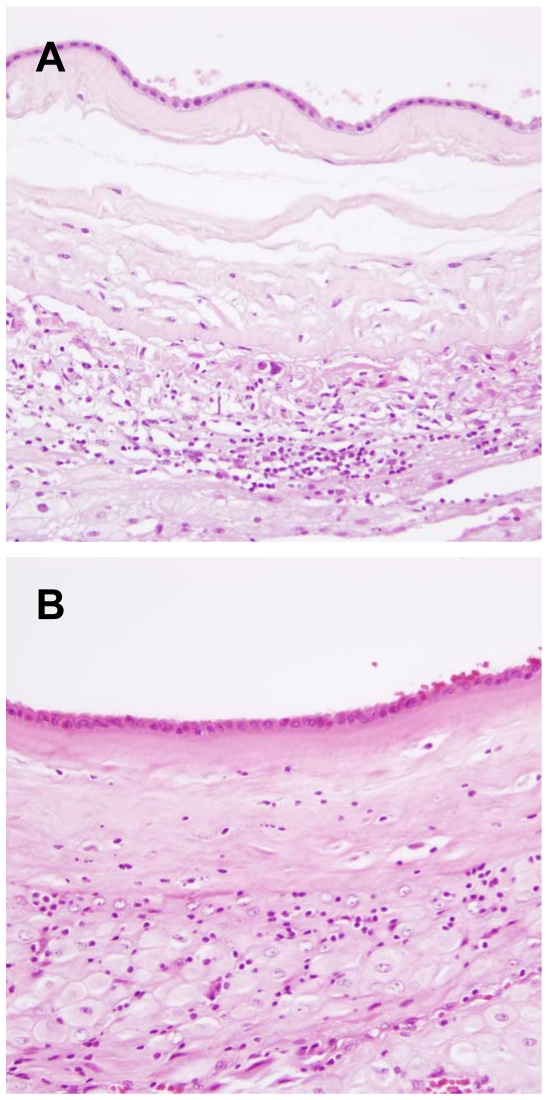
Histological features of chronic chorioamnionitis. (A) A case of spontaneous preterm birth showing destruction of the chorionic trophoblast layer by infiltrating lymphocytes. (B) In another case of spontaneous preterm birth, lymphocytic infiltration extended to the chorioamniotic connective tissue layer. Original magnification X200.

Table 1 shows the demographics of the study population. There were significant differences in the proportion of cases with placental changes (maternal vascular underperfusion and chronic chorioamnionitis) between cases with normal term birth and spontaneous preterm birth (p<0.01, for each). Differences were observed in maternal age, gravidity, parity, and frequencies of cesarean delivery (p<0.05, for each). Chronic chorioamnionitis is histologically characterized by maternal T cell infiltration in the chorionic trophoblast layer or chorioamniotic connective tissue layer. Representative histological features are illustrated in Figure 1.

**Table 1:** Demographics of study populations.

	Term birth	Spontaneous preterm birth	
	n = 140	n = 140	p
Maternal age (years)*	27.4±0.5	26.0±0.7	0.036
Primigravida (%)	15.7 (22/140)	42.9 (60/140)	<0.001
Nulliparous (%)	17.9 (25/140)	47.9 (67/140)	<0.001
Gestational age at delivery (weeks)*	39.5±0.1	34.0±0.2	<0.001
Cesarean delivery (%)	50.0 (70/140)	22.1 (31/140)	<0.001
Birth weight (g)*	3416.7±26.7	2377.4±47.5	<0.001
Baby gender (male sex) (%)	55.0 (77/140)	67.1 (94/140)	0.037
Placental pathology			
Acute chorioamnionitis (%)	12.9 (18/140)	18.6 (26/140)	NS
Maternal underperfusion (%)	2.1 (3/140)	12.9 (18/140)	0.001
Fetal vascular thrombo-occlusive disease (%)	6.4 (9/140)	6.4 (9/140)	NS
Villitis of unknown etiology (%)	15.7 (22/140)	22.1 (31/140)	NS
Chronic chorioamnionitis (%)	13.6 (19/140)	40.7 (57/140)	<0.001

*Mean±SEM.

**Figure 2:**
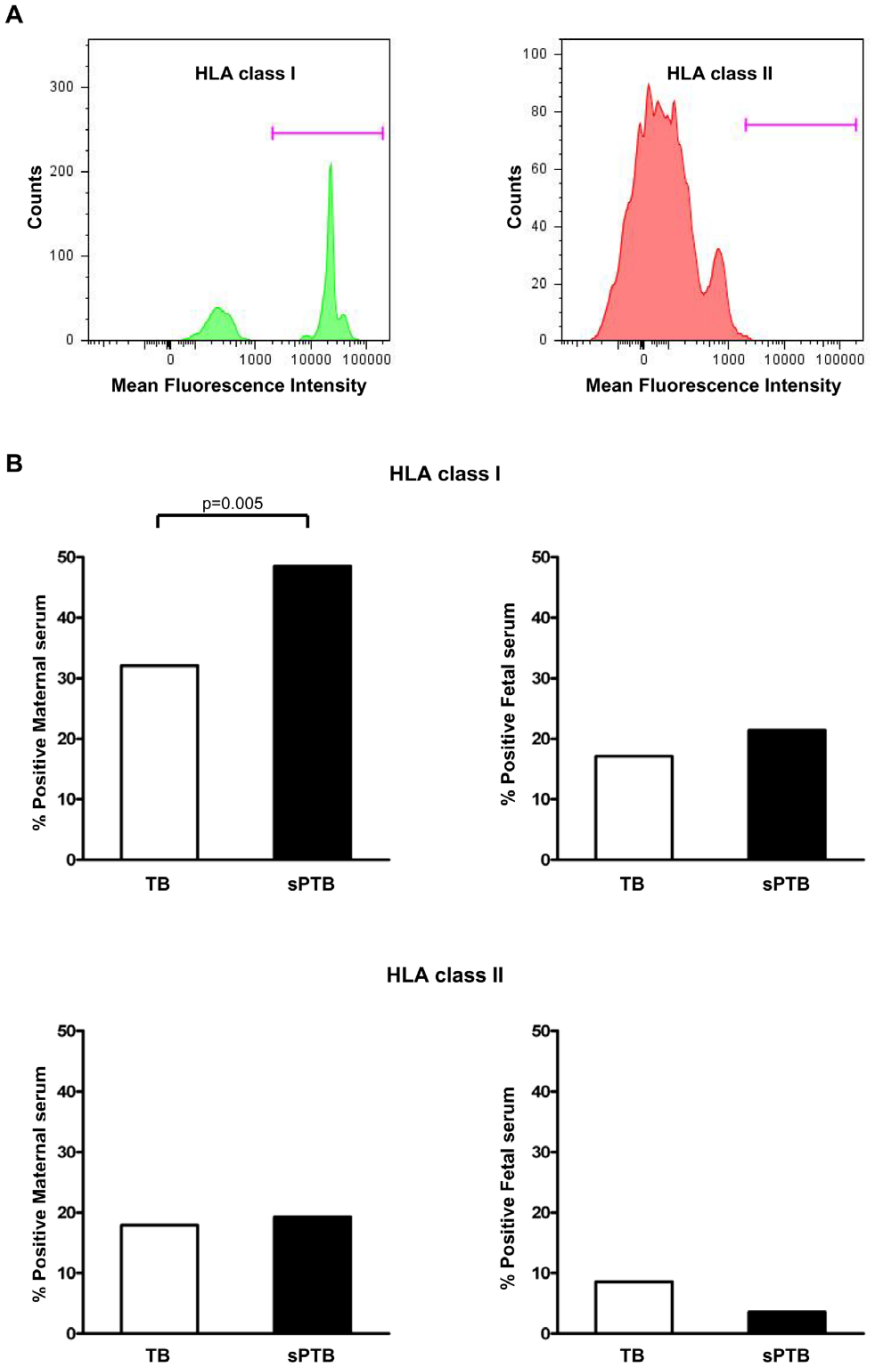
Panel-reactive anti-HLA seropositivity in maternal and fetal sera. (A) A representative case showing the presence of anti-HLA class I antibodies and the absence of anti-HLA class II antibodies in maternal serum with FlowPRA®-I and -II screening tests, followed by flow cytometry. (B) Spontaneous preterm birth cases have a higher maternal anti-HLA class I seropositivity than normal term birth cases (p<0.01). *TB*, normal term birth; *sPTB*, spontaneous preterm birth.

Figure 2A illustrates the presence of anti-HLA class I antibodies detected by flow cytometry in maternal serum of a representative case. To determine whether anti-HLA antibodies play a role in spontaneous preterm birth, anti-HLA seropositivity of women presenting with spontaneous preterm birth was compared to cases of normal term birth (Figure 2B). Spontaneous preterm birth had a higher maternal anti-HLA class I seropositivity than normal delivery at term (48.6% vs. 32.1%, p = 0.005). There was a significant difference in maternal anti-HLA class I seropositivity among cases classified in detail according to their clinical diagnosis (cases with term in labor, 31.4%; cases with term not in labor, 32.9%; cases with preterm labor and intact membranes, 52.9%; cases with preterm premature rupture of membranes, 44.3%; p = 0.002).

**Figure 3:**
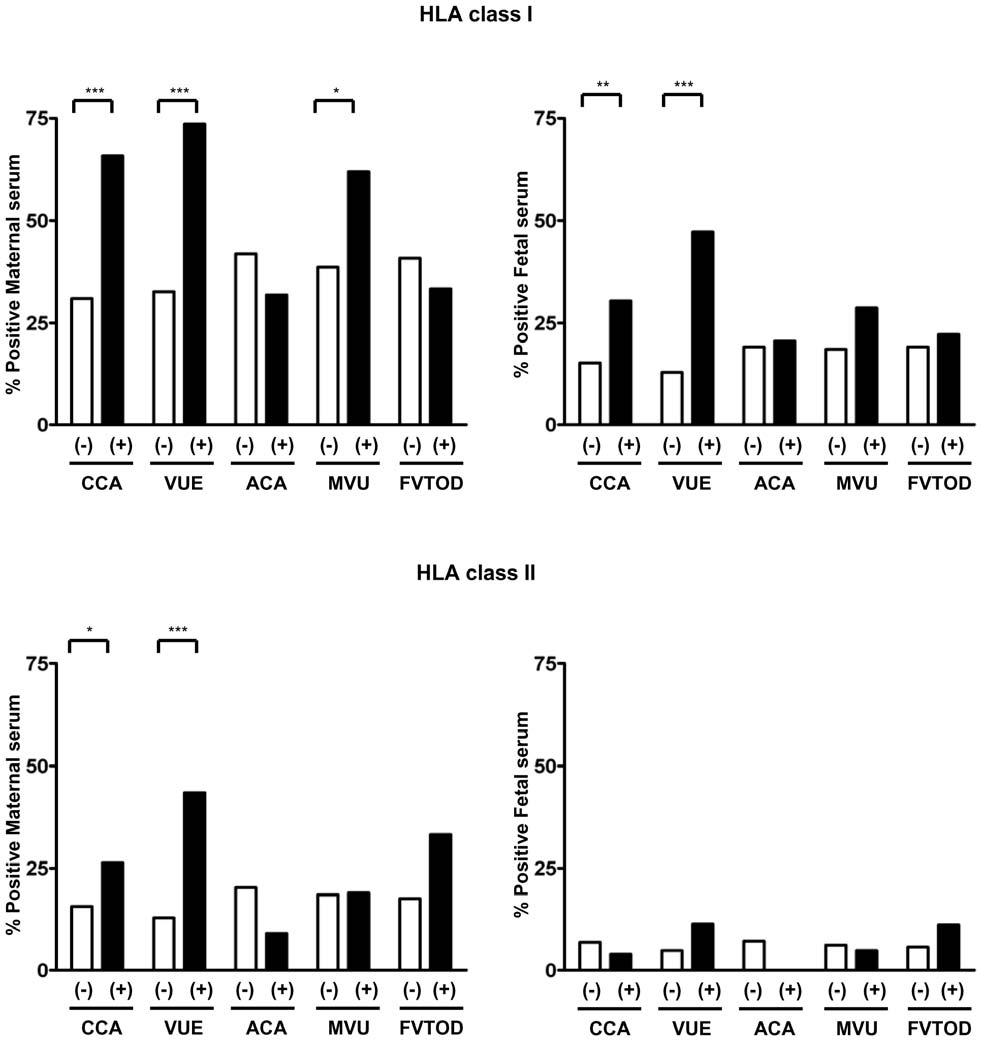
Comparisons of maternal and fetal panel-reactive anti-HLA class I and II seropositivity according to presence or absence of placental pathologic lesions. Maternal anti-HLA class I and class II seropositivity and fetal anti-HLA class I seropositivity is higher in chronic chorioamnionitis cases than those without chronic chorioamnionitis (p<0.05, for each). Similarly, maternal anti-HLA class I and class II seropositivity and fetal anti-HLA class I seropositivity is higher in villitis of unknown etiology cases than those without (p<0.001, for each). Such differences in anti-HLA seropositivity are not found in the cases with acute chorioamnionitis, maternal vascular underperfusion, and fetal vascular thrombo-occlusive disease except for maternal anti-HLA class I seropositivity in cases with maternal vascular underperfusion (p<0.05). *CCA*, chronic chorioamnionitis; *VUE*, villitis of unknown etiology; *ACA*, acute chorioamnionitis; *MVU*, maternal vascular underperfusion; *FVTOD*, fetal vascular thrombo-occlusive disease. *, p<0.05; **, p<0.01; ***, p<0.001.

Figure 3 shows the relationship between anti-HLA seropositivity and placental pathology. When anti-HLA seropositivity was compared according to placental pathology, maternal anti-HLA class I and class II seropositivity and fetal anti-HLA class I seropositivity were higher in chronic chorioamnionitis cases than in those without chronic chorioamnionitis (for maternal anti-HLA class I antibodies: 65.8% vs. 30.9%, p<0.001; for maternal anti-HLA class II antibodies: 26.3% vs. 15.7%, p = 0.042; for fetal anti-HLA class I antibodies: 30.3% vs. 15.2%, p = 0.004). Similarly, cases with villitis of unknown etiology had higher maternal and fetal anti-HLA class I and class II seropositivity than those without (for maternal anti-HLA class I antibodies: 73.6% vs. 32.6%, p<0.001; for maternal anti-HLA class II antibodies: 43.3% vs. 12.8%, p<0.001; for fetal anti-HLA class I antibodies: 47.2% vs. 12.8%, p<0.001). For other placental pathologic lesions consistent with amniotic fluid infection, maternal vascular underperfusion, and fetal vascular thrombo-occlusive disease, anti-HLA class I or class II seropositivity was not different, with the exception that maternal anti-HLA class I seropositivity was higher in cases with maternal vascular underperfusion than in those without (p = 0.036).

To determine the relative effects of clinical variables and placental pathology on maternal and fetal anti-HLA seropositivity, logistic regression analysis was performed. After adjusting for five placental pathologic findings and clinical variables (e.g., gestational age at delivery, maternal age, fetal gender, gravidity), chronic chorioamnionitis, villitis of unknown etiology, and previous pregnancy increased maternal anti-HLA class I seropositivity (for chronic chorioamnionitis: OR 3.84, 95% CI 1.97–7.48, p<0.001; for villitis of unknown etiology: OR 5.22, 95% CI 2.47–11.03, p<0.001; for previous pregnancy: OR 2.63, 95% CI 1.20–5.78, p = 0.016). Although the effects of chronic chorioamnionitis on fetal anti-HLA class I antibody seropositivity did not reach statistical significance (p = 0.102), villitis of unknown etiology increased maternal anti-HLA class II seropositivity and anti-HLA class I and II seropositivity in the fetus (p<0.05, for each).

The results from within-group comparisons were also consistent. Maternal anti-HLA class I seropositivity was higher in chronic chorioamnionitis cases than in those without chronic chorioamnionitis both in normal term births (63.2% vs. 27.3%, p = 0.002) and spontaneous preterm births (66.7% vs. 36.1%, p<0.001). Maternal anti-HLA class II seropositivity was higher in cases of normal term birth with chronic chorioamnionitis than in cases without (42.1% vs. 14.0%, p = 0.007). Fetal anti-HLA class I seropositivity was also higher in spontaneous preterm birth cases with chronic chorioamnionitis than in those without this lesion (33.3% vs. 13.3%, p = 0.004).

**Figure 4:**
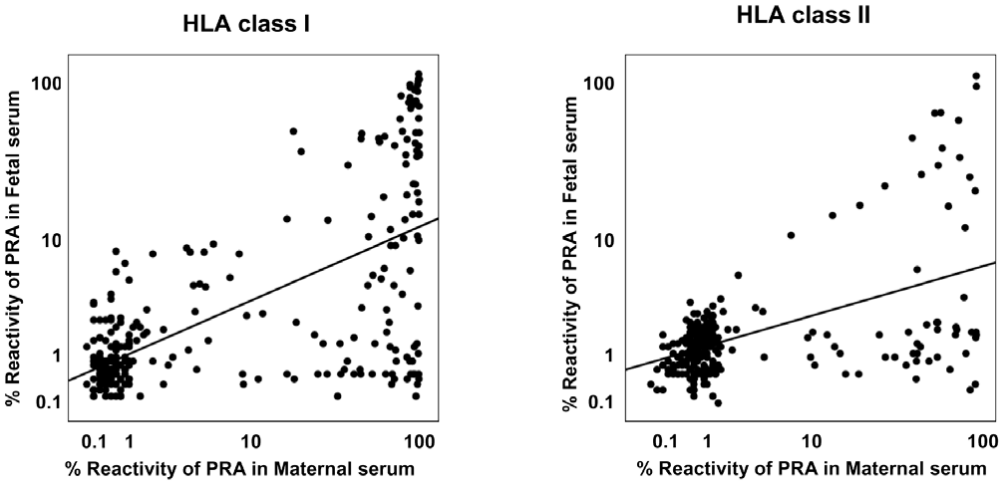
Correlation between maternal and fetal anti-HLA class I and class II antibodies. The panel reactivity of maternal anti-HLA antibodies is associated with that of fetal anti-HLA antibodies (for anti-HLA class I antibodies: r = 0.578, p<0.001; for anti-HLA class II antibodies: r = 0.358, p<0.001). *PRA*, panel-reactive anti-HLA antibodies.

Figure 4 describes good correlations between the panel reactivity of maternal and fetal anti-HLA class I and II antibodies (for anti-HLA class I antibodies: r = 0.578, p<0.001; for anti-HLA class II antibodies: r = 0.358, p<0.001). The correlation between the panel reactivity of maternal and fetal anti-HLA class I and II antibodies was significant in each group of normal term births and spontaneous preterm births, respectively (p<0.01, for each).

**Figure 5:**
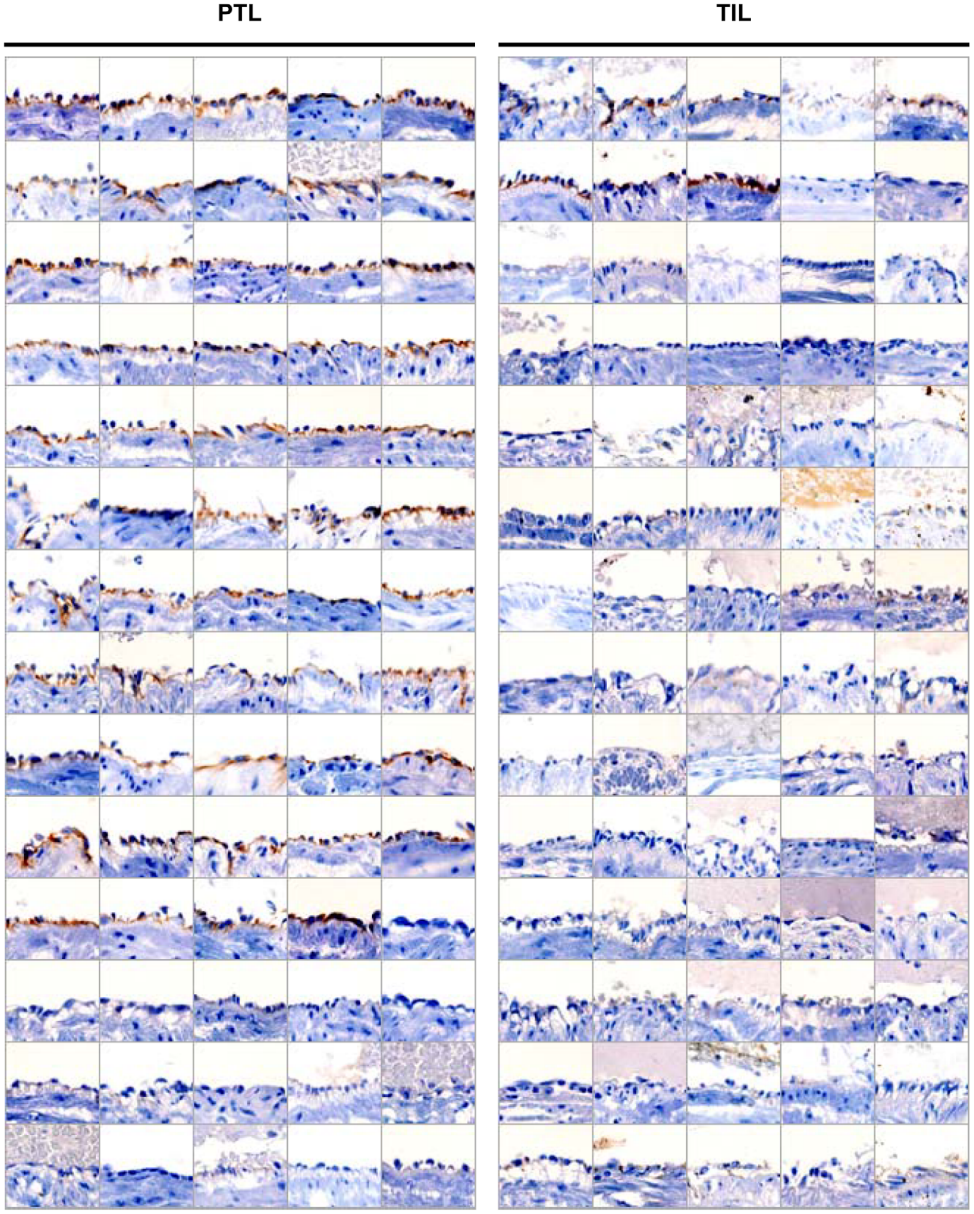
C4d deposition on fetal umbilical vein endothelium by immunohistochemistry. C4d immunoreactivity on fetal umbilical vein endothelium is found in 54 out of 70 cases with preterm labor and intact membranes followed by preterm birth. However, there are only eight C4d positive cases among 70 cases with normal term labor (77.1% vs. 11.4%; p<0.001). *PTL*, preterm labor and intact membranes and preterm birth; *TIL*, term labor and normal term birth.

The presence of alloimmune anti-HLA antibodies in fetal circulation led us to investigate whether there is antibody-dependent complement activation in the fetus as a demonstration of antibody-mediated allograft rejection. Immunohistochemical staining for C4d was performed using umbilical cord sections obtained from term in labor (n = 70) and preterm labor (n = 70) cases. Figure 5 shows C4d deposition on umbilical vein endothelium. Distinct C4d immunoreactivity confined to umbilical vein endothelium, but not in umbilical arterial endothelium, was found in 77.1% (54/70) of preterm labor cases. In contrast, C4d immunoreactivity was observed only in 11.4% (8/70) of term labor cases, showing stark contrast with preterm labor cases (p<0.001). In addition, C4d deposition in umbilical vein endothelium was significantly associated with the presence of chronic chorioamnionitis (75.9% vs. 36.0%, p<0.001), and also tended to be more frequent in anti-HLA class I seropositive fetuses compared to seronegative fetuses (60.0% vs. 40.0%, p = 0.051).

Logistic regression analysis was done to determine the respective risk of placental pathology, maternal and fetal anti-HLA seropositivity, and C4d immunoreactivity for preterm labor. The analysis showed that chronic chorioamnionitis (OR: 6.10, 95% CI 1.29–28.83, p = 0.023), maternal vascular underperfusion (OR: 21.32, 95% CI 1.63–278.75, p = 0.02), maternal anti-HLA class I seropositivity (OR: 5.90, 95% CI 1.60–21.83, p = 0.008), and C4d deposition on umbilical vein endothelium (OR: 36.19, 95% CI 11.42–114.66, p<0.001) increased the odds of preterm labor and preterm birth.

## Discussion

The principal findings of this study are: (1) maternal anti-HLA class I seropositivity in spontaneous preterm births is higher than that of normal term births; (2) mothers with chronic chorioamnionitis and villitis of unknown etiology have significantly higher anti-HLA class I seropositivity than those without these lesions; (3) C4d immunoreactivity on umbilical vein endothelium, evidence of the activation of the classical pathway of the complement system, is a common feature of preterm but not term labor; and (4) chronic chorioamnionitis, maternal anti-HLA class I seropositivity, and umbilical vein endothelial C4d increase the odds ratio of spontaneous preterm birth.

The positive rate of anti-paternal cytotoxic antibody testing against a partner's lymphocytes increases as a function of gestational age in normal pregnant women [22]. This study demonstrates the potential importance of maternal anti-fetal antibody-mediated rejection in human spontaneous preterm birth for the first time. It also establishes a solid and novel link between the presence of maternal antibody-mediated rejection and chronic chorioamnionitis, a major pathology in spontaneous preterm birth [18]. Chronic chorioamnionitis and villitis of unknown etiology are associated with systemic changes in T cell chemokines in the fetus [17], [18]. However, the precise mechanisms for this association between these placental inflammatory lesions and fetal systemic inflammatory response have remained elusive. Maternal IgG can cross the placenta [33], and this humoral variety of alloimmune reaction, in essence, would have a greater chance of inducing a systemic reaction in the fetus. A murine model of ^125^I-labeled monoclonal anti-paternal MHC antibody injection into pregnant mice clearly demonstrated accumulation of the antibodies in various fetal tissues, particularly in blood, thymus, and liver [34]. Accumulation of intact antibodies in fetal tissues persisted up to three days after injection, while those were eliminated in the mother. In humans, examples of neonatal alloimmune thrombocytopenia due to anti-HLA antibodies provide compelling evidence that anti-HLA alloimmunity could have systemic consequences in the fetus [29], [35], [36]. Of note, Althaus et al described an association between chronic villitis and neonatal alloimmune thrombocytopenia [37]. When they compared the placental findings of 14 pregnant women who were treated or untreated with antepartum intravenous immunoglobulin, chronic villitis was found in 83% (5/6) of the untreated group while none of the treated cases (0/8) had chronic villitis. Gu et al also showed that patients with active immune thrombocytopenia have elevated plasma CXCL10 concentration and higher CXCL10 mRNA expression in peripheral blood mononuclear cells compared to normal healthy subjects [38]. These observations support a link between anti-HLA antibodies and chronic chorioamnionitis and villitis of unknown etiology.

Anti-HLA class I seropositivity, higher than anti-HLA class II seropositivity observed in this study, is intriguing. Sensitization to allo-HLA antigens can follow previous pregnancy, blood transfusion, and organ transplantation [16], [39]. Possible explanations for maternal sensitization against fetal HLA antigens would be: 1) recognition of fetal HLA antigens expressed in the remnants of trophoblasts or in the chorioamniotic membranes by maternal effector cells during the cleaning up of the uterine cavity after delivery [40], 2) feto-maternal hemorrhage [41], [42], and 3) fetal cell trafficking into the mother [43]. Direct or indirect interactions between fetal and maternal cells occur in the chorionic villi and the chorioamniotic membranes [44], and evidence for trafficking of fetal antigens to maternal secondary lymphoid organs has been demonstrated [43]. As HLA class I antigens are more ubiquitously expressed in various cell types compared to HLA class II antigens, this could explain why anti-HLA class I seropositivity is higher in pregnant women. It is noteworthy that HLA-C but not HLA class II expression can be detected in the chorionic trophoblasts [44], [45]. Interestingly, the significance of alloimmune antibodies has been studied largely in recurrent miscarriage, yet it remains controversial [21]–[27]. While earlier studies have reported that the absence or low titer of anti-paternal cytotoxic antibodies are associated with recurrent miscarriages [25], [26], a recent study does not support an association between unexplained recurrent miscarriage and anti-HLA antibodies [24]. Therefore, systematic comparative analysis of spontaneous preterm birth and spontaneous abortion would be an important subject of future investigations.

A stark difference in umbilical venous endothelial C4d immunoreactivity between term and preterm birth cases is also robust. C4d deposition on endothelium is a surrogate marker of antibody-mediated rejection in organ transplantation [46]. In the current study, we determined anti-HLA antibodies in maternal and fetal sera and C4d deposition on umbilical vein endothelium for the evaluation of antibody-mediated rejection, and both of them increase the odds ratio of the occurrence of preterm birth.

Recently, a National Institutes of Health consensus conference proposed four stages of chronic antibody-mediated rejection: stage I) latent humoral response (the presence of circulating alloantibody); stage II) silent humoral rejection (stage I plus detection of C4d); stage III) subclinical humoral rejection (stage II plus tissue injury or pathological change in graft); and stage IV) clinical humoral rejection (stage III plus graft dysfunction) [47], [48]. Anti-HLA antibodies in maternal and fetal sera and C4d deposition in umbilical vein endothelial cells in the current study could be considered stage I or II antibody-mediated rejection, while chronic chorioamnionitis, villitis of unknown etiology, and spontaneous preterm birth could be analogous to stage III or IV antibody-mediated rejection. While anti-HLA seropositivity and C4d show good correlations in organ transplantation [49], [50], we found a marginal association between fetal anti-HLA class I antibodies and C4d deposition on umbilical vein endothelium in this study. Therefore, it would be worthwhile to study roles of other non-HLA antibodies such as anti-endothelial antibodies and non-classical MHC class I-related chain A (MICA)-specific alloantibodies in C4d deposition in spontaneous preterm birth [51]–[54].

During pregnancy, women are also primed to minor histocompatibility (H) antigens such as HA-1, HA-2, and HY antigens with subsequent generation of H antigen-specific CD8+ cytotoxic T cells [55], [56]. Anti-HY immunity was also shown to be associated with recurrent miscarriages and placental abruption [57]. Of note, a recent study of 66,387 women demonstrated a higher rate of preterm delivery in male fetuses due to an increase in spontaneous preterm labor and preterm prelabor rupture of membranes [58].

There are limitations of this cross-sectional study. We could not determine the potential impact of dynamic changes in the anti-HLA antibodies status before and during pregnancy on the development of spontaneous preterm birth, and further studies on a longitudinal basis will be needed to address this issue. Another limitation is that this clinical analysis does not provide a direct mechanistic link between anti-fetal rejection and the clinical presentation: preterm labor and preterm prelabor rupture of membranes. This study was also restricted to the analysis of spontaneous preterm births. Because medically indicated preterm birth due to conditions such as preeclampsia and fetal growth restriction comprises a substantial proportion of preterm births and because chronic chorioamnionitis is also observed in these pregnancy complications, future studies on the antibody-mediated rejection in the setting of indicated preterm birth are required.

Collectively, we propose that a subset of spontaneous preterm birth has a signature of maternal anti-fetal rejection, in which the phenotypic complex of cellular rejection is comprised of chronic chorioamnionitis and villitis of unknown etiology coupled with graft-versus-host disease of the fetus [17], [18]. The phenotype of antibody-mediated rejection is generation of anti-paternal/fetal antibodies in the mother and fetal alloimmune reaction due to these antibodies and complement activation. Although human pregnancy displays many analogous features to allograft transplantation, it also poses a very unique biological situation because the fetus is another host with isolated circulation from the mother. Novel findings herein have profound implications in understanding the biology of preterm birth and developing preventive and therapeutic modalities.
